# Effects of Telemedicine on Dysphagia Rehabilitation in Patients Requiring Home Care: A Retrospective Study

**DOI:** 10.1007/s00455-025-10844-0

**Published:** 2025-06-07

**Authors:** Rieko Moritoyo, Kazuharu Nakagawa, Kanako Yoshimi, Kohei Yamaguchi, Yuki Nagasawa, Ryosuke Yanagida, Koji Hara, Haruka Tohara

**Affiliations:** Division of Gerontology and Gerodontology, Department of Dysphagia Rehabilitation, Graduate School of Medical and Dental Sciences, Institute of Science Tokyo (Formerly Tokyo Medical and Dental University), 1-5-45 Yushima, Bunkyo-ku, Tokyo, 113-8510 Japan

**Keywords:** Telemedicine, Dysphagia, Home-visit medical care, Dysphagia rehabilitation, Remote healthcare, Swallowing function

## Abstract

This study investigated telemedicine as an alternative to home-visit medical care (HMC) when HMC for patients with dysphagia was suspended. This retrospective study assessed whether telemedicine reduced adverse events compared to suspending care during the initial 3 months of the coronavirus pandemic. Seventy-six HMC patients were enrolled. Those who received telemedicine formed the telemedicine group (TG), and those who declined comprised the suspended group (SG). Baseline data and adverse events, including whole-body and dysphagia-related adverse events, were analyzed using the Mann–Whitney U-test, Fisher’s exact test, and binomial logistic regression. Of the 76 patients, 20 were in TG and 56 in SG. Telemedicine consultations’ frequency was 1–3. Significant baseline differences occurred in the Charlson Comorbidity Index (CCI) and caregiver type. In the TG and SG, 0% and 12.5% of patients experienced whole-body adverse events and 10.0% and 33.9% had dysphagia-related adverse events, respectively. Dysphagia-related adverse events were significantly lower in TG (p = 0.046). Telemedicine was significantly associated with fewer dysphagia-related adverse events after adjusting for age, CCI, and Dysphagia Severity Scale (p = 0.040). Telemedicine effectively supplemented in-person dysphagia rehabilitation, enabling continued monitoring and reducing complications, although patient self-selection and caregiver support may have influenced outcomes.

## Introduction

The novel coronavirus disease 2019 (COVID-19) posed a severe threat worldwide and was officially declared a pandemic by the World Health Organization on March 11, 2020. Adequate infection control measures are essential in dysphagia rehabilitation owing to the high risk of droplet infections during assessments and examinations.

At the Department of Dysphagia Rehabilitation, Institute of Science Tokyo Hospital, a tertiary care hospital in Tokyo, Japan, dentists provide swallowing function assessments and dysphagia rehabilitation through outpatient and home-visit medical care (HMC) services. Hospital care was reduced to the minimum necessary in 2020 to limit the spread of the COVID-19 pandemic, leading to the suspension of home visits. During this period, dysphagia rehabilitation continued via information and communication technology (ICT)-based telemedicine for patients who requested it. This approach was particularly vital for individuals with underlying medical conditions and older adults, as it minimized exposure to large groups and reduced the risk of COVID-19 and other infectious diseases. Telemedicine facilitated medical care when face-to-face treatment was not feasible. In this study, telemedicine refers to medical care conducted using ICT to connect hospital-based dentists with patients and caregivers at home or with healthcare professionals regularly involved in home-based interventions.

In fact, telemedicine has gained significant popularity since the COVID-19 pandemic, with clinician questionnaire surveys reporting positive results regarding convenience, accessibility, and cost-effectiveness [1]. Even before the pandemic, patients with dysphagia received clinical swallowing assessments and treatment monitoring from specialist clinicians, with high levels of satisfaction reported by both patients and clinicians regarding service quality and cost [2, 3]. The reliability and validity of telemedicine assessments of swallowing function have been demonstrated in several prospective intervention studies. High concordance rates for recommended food textures and other factors have been observed in patients with acute stroke and mild dementia when swallowing function assessments were conducted by skilled evaluators in face-to-face and online settings [4, 5]. Retrospective observational studies have also examined patient factors that could act as barriers to online swallowing function assessments, such as speech and language disorders, hearing impairment, motor impairment, and behavioral and emotional challenges [6]. Moreover, causative conditions of dysphagia have been shown to influence telehealth swallow therapy attendance. Patients with dysphagia owing to cancer or muscle tension are less likely to engage in telemedicine than those with globus, GERD/LPR, post-surgical effects, or unspecified dysphagia [7]. Effective management of dysphagia via telehealth depends on critical technological infrastructure, including reliable internet access, specialized equipment, and user-friendly platforms. However, significant challenges persist, such as inadequate infrastructure, limited patient digital literacy, and cost-related barriers, as highlighted in a survey conducted among specialized medical professionals [8].

Specific validation of telemedicine-based dysphagia rehabilitation is gradually increasing. In airway protection training using telemedicine, expiratory muscle strength training has been shown to improve maximum expiratory pressure, and cough skill training increases peak expiratory flow rate. The time required for a session to be feasible has also been verified [9]. A study reporting three cases demonstrated that tele-dysphagia interventions, such as cyclic ingestion, chin-down, and head-turn strategies, had effects comparable to those of in-person therapy, with participants achieving high goal accuracy rates (83–90%) [10]. Telepractice therapy using the SwallowIT application has shown clinical equivalence to face-to-face therapy in swallowing, nutritional, and functional outcomes for patients with head and neck cancer undergoing (chemo)radiotherapy while offering cost savings and higher patient satisfaction than self-directed therapy [11, 12]. These interventional studies highlight the usefulness and effectiveness of telemedicine for swallowing training instruction but do not address its impact on preventing adverse events related to decreased swallowing function or long-term outcomes.

Although telemedicine is becoming established as a new practice technique in dysphagia rehabilitation [13], there are no studies, to the best of our knowledge, that have statistically evaluated the benefits of telemedicine dysphagia rehabilitation in reducing the incidence of adverse events in patients with dysphagia requiring home care. In a cross-sectional survey of older adults recovering at home, a decline in dietary form within 1 year, indicating reduced swallowing function, was associated with increased risks of hospitalization (hazard ratio: 6.35) and death (hazard ratio: 3.76) [14]. However, infectious disease outbreaks or other factors may preclude continuous assessment of swallowing function through face-to-face medical care. Therefore, continued assessment and intervention of swallowing function are important to prevent adverse events, including aspiration pneumonia [15] and choking [16].

This study aimed to retrospectively investigate the reduction of adverse events in patients with dysphagia unable to receive face-to-face visits at home or in long-term care facilities. We compared patients who self-selected to continue care via telemedicine with those who temporarily suspended care. Additionally, we examined factors influencing the reduction in adverse events. We hypothesized that during unavoidable suspensions of HMC, patients who received telemedicine-based dysphagia rehabilitation as an alternative to face-to-face care would experience fewer adverse events than those whose dysphagia care was suspended.

## Methods

### Participants

Patients with dysphagia aged ≥ 20 years, who were regularly visited at home or in a nursing home by dentists from our department every 1–3 months, were included in this retrospective study. Dysphagia was evaluated by dentists in our department. Patients with medical records from the 3 months preceding the declaration of the state of emergency on April 7, 2020, and with appointments scheduled between April 7 and June 15, were selected. Before the suspension of HMC, swallowing endoscopy was performed every 1–3 months during face-to-face visits. Based on examination results, patients were instructed on the appropriate dietary form, swallowing training, and environmental settings for eating and training. The frequency of face-to-face visits was determined by the dentist in charge based on the patient’s condition. After the suspension of face-to-face visits, telemedicine was explained to patients via telephone and provided if they or their family members opted for it. The dentist in charge also determined the frequency of telemedicine consultations, and all patients who received telemedicine complied. Patients who declined telemedicine had their treatment suspended until June 15, 2020, when restrictions on medical treatment at our hospital were lifted, and face-to-face care was resumed. The exclusion criteria included patients who, after the lifting of restrictions, declined to resume HMC owing to concerns about infection control.

### Intervention

Telemedicine was implemented from April 7, 2020, to June 15, 2020, during the suspension of the hospital’s clinic system. Patients and the hospital were connected via mobile phones with camera functions, PCs, tablets, and other information and communication devices. An online information system called YaDoc (Integrity Healthcare Co., Ltd., Tokyo, Japan), a telemedicine application that encrypts medical information and communication, was used. On the patient side, connections were facilitated by patients themselves or telemedicine supporters, such as family members, caregivers, or healthcare professionals. The YaDoc application was selected for its user-friendly interface and robust focus on protecting personal information. The dentist provided instructions on downloading and using the YaDoc application to the patient or telemedicine supporters via phone or email. No prior training or preparation was required for either dentists or patients in using YaDoc. No pilot test or trial period was needed, and no modifications were made to the YaDoc application during the study. Before starting the telemedicine consultation, the dentist and patient tested the connection to ensure the system was functioning properly. Each telemedicine session lasted approximately 30 min, during which the dentist interviewed the patient and observed their eating habits. Based on the interview findings and the patient’s eating habits, the dentist provided guidance on compensatory techniques, as well as direct and indirect therapies. Specifically, compensatory techniques included adjustment of dietary forms, eating environments, and posture during meals. Direct swallowing therapy involved various exercises such as swallowing practice with thickened water or jelly and chewing practice using snack foods. Indirect swallowing therapy included oral motor exercises, strengthening exercises for swallowing-related muscles such as the suprahyoid muscles, and respiratory rehabilitation. Additionally, instructions were provided on nutritional management, oral hygiene, and the use of oral protective mouthpieces. Eating habits were classified into the following three categories: independent oral intake (self-intake of meals), assisted oral intake (with caregiver support), and oral intake practice (small quantities of food consumed for practice when primary nutrition was provided non-orally). The content of each group’s intervention is categorized into Evaluation/Assessment and Instructions/Treatment, as detailed in Table 1. The frequency of online visits was determined using the same criteria as for the previously conducted home visits. The dentist made decisions comprehensively based on clinical findings, including the severity of dysphagia, the presence of aspiration or laryngeal penetration observed during video endoscopy examination, the risk of aspiration pneumonia, the patient’s nutritional status, and the level of concern expressed by the patient or their family regarding swallowing function. When clinical findings indicated a higher risk, the frequency of visits was set at 1–2 times per month. If the clinical condition was stable, the interval between visits was extended to more than 2 months. Telemedicine was provided by the same dentist who had been conducting face-to-face visits before the COVID-19 pandemic. All participating dentists had at least 10 years of clinical experience in dysphagia rehabilitation.

**Table 1:** Contents of telemedicine consult

Eating habits (n = 20)	Evaluation/Assessment (with duplicates)	Instructions/Treatment (with duplicates)
Self-intake of meals (7)	Dietary form and intake (7)	Indirect swallowing training (5) Eating methods (2) Maintenance of the status quo (2) Adjustment of eating environment (1)
Assisted oral intake of meals (6)	Dietary form and intake (6) Nutritional status (3) Oral hygiene status (1)	Adjustment of eating environment (2) Nutritional management (2) Maintenance of the status quo (2) Guidance for meal assistance (1) Adjustment of dietary form (1) Oral hygiene guidance (1) Responding to choking (1)
Practice of oral intake (7)	Direct swallowing therapy by child/parent/sibling (4) Direct swallowing therapy by spouse (3) Nutritional status (1) Usage of oral protective mouthpieces (1)	Guidance on direct swallowing therapy methods (5) Addition of direct swallowing therapy menu (2) Nutritional management (1) Maintenance of the status quo (1)

Self-intake of meals: When the patient eats independently; Assisted intake of meals: When the patient eats with assistance from a caregiver; Practice of oral intake: When the patient consumes small amounts of food orally for practice, not as a nutritional measure. “With duplicates” indicates that the number reflects instances where patients received multiple types of Evaluation/Assessment or Instructions/Treatment; therefore, a single patient may be counted more than once

### Outcomes

Basic participant information, including age, sex, body mass index (BMI), primary disease, medical history, history of aspiration pneumonia, activity status, and the Charlson Comorbidity Index (CCI) [17], was collected from medical records as of April 1, 2020. The Barthel Index (BI) score [18] was obtained to assess the patient’s activity status. Oral intake status was evaluated using the Functional Oral Intake Scale (FOIS) [19], which has the following seven levels: 1 = no oral intake; 2 = tube-dependent with minimal/inconsistent oral intake; 3 = tube supplements with consistent oral intake; 4 = total oral intake of a single consistency; 5 = total oral intake of multiple consistencies requiring special preparation; 6 = total oral intake with no special preparation but avoidance of specific foods or liquids; and 7 = total oral intake with no restrictions [19]. Baseline dysphagia severity was assessed using the Dysphagia Severity Scale (DSS), based on swallowing endoscopy performed within 3 months before the suspension of HMC. The DSS is a seven-point scale, with lower scores indicating more severe dysphagia [20]. Data were also collected on the primary provider of dysphagia care and whether healthcare professionals, including home care nurses, speech-language pathologists, and dental hygienists, provided interventions for dysphagia. Additionally, the number of days between the last face-to-face medical care before the suspension of home visits and the resumption of face-to-face care was calculated.

We retrospectively assessed the number of adverse events related to whole-body conditions or dysphagia that occurred between April 7, 2020, and June 15, 2020, during the suspension of our clinic system and home visits. Adverse events related to whole-body conditions were defined as follows: (1) death during the suspension period, regardless of the cause, and (2) hospitalization during the suspension period, regardless of the underlying disease. Adverse events related to dysphagia were defined as follows: (1) aspiration pneumonia, diagnosed by the attending physician; (2) choking, characterized by symptoms of airway obstruction with food; and (3) decline in swallowing function, identified by coughing when food or liquids approached the airway, prolonged mealtime exceeding 10 min since the last face-to-face visit, or weight loss of > 5% of baseline body weight owing to reduced food intake. Choking and decline in swallowing function were directly assessed by the dentist through interviews with the patient or caregiver during the first resumed visit after June 16, 2020. If a patient’s medical record contained information meeting any of these criteria, it was classified as a dysphagia-related adverse event.

### Statistical Analysis

The participants were categorized into the following two groups: those who received telemedicine (telemedicine group: TG) and those who did not and were suspended from receiving HMC (suspended group: SG). Statistical analysis was conducted to determine whether differences existed between the TG and SG regarding basic participant information and the incidence of adverse events. Continuous variables, including age, BMI, BI, FOIS, DSS, and the number of days of suspended face-to-face medical care, were analyzed using the Mann–Whitney U or t-test, with normal distribution confirmed using the Shapiro–Wilk test. Categorical variables, such as sex, medical history, history of aspiration pneumonia, and incidence of adverse events, were analyzed using Fisher’s exact test.

Factors associated with dysphagia-related adverse events were also examined. Patient characteristics were compared based on the presence or absence of dysphagia-related adverse events. Using univariate analysis, dysphagia-related adverse events were set as the dependent variable, and all survey items were treated as independent variables to calculate crude odds ratios and 95% confidence intervals. Binomial logistic regression analysis was performed to adjust for confounding factors, with dysphagia-related adverse events classified into the following two groups: with and without dysphagia-related adverse events. Telemedicine implementation, age, CCI, and DSS were selected as independent variables. Age was adjusted as it is associated with dysphagia [21], and CCI was included owing to significant differences observed between TG and SG. DSS, reflecting the clinical severity of dysphagia, was incorporated as a predictor of adverse events. DSS scores (1–4: aspiration present; 5–7: aspiration absent) were divided into these categories owing to the significant impact of aspiration on dysphagia outcomes. Statistical significance was set at 5%. Analyses were conducted using IBM SPSS Statistics for Windows, version 28.0.1.1 (IBM Corp, Armonk, NY, USA).

## Results

### Participant Characteristics

**Fig. 1:**
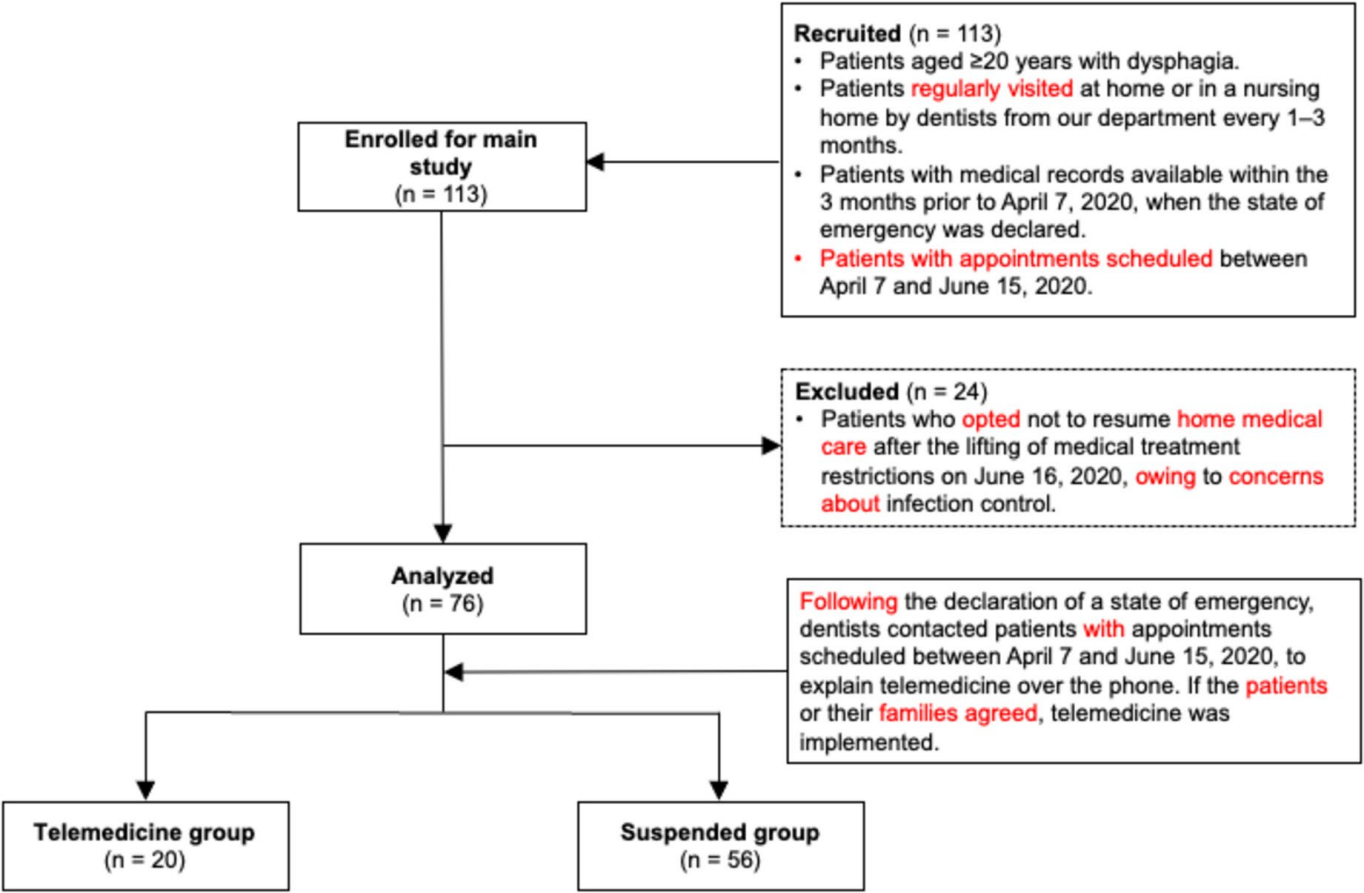
Flowchart showing the recruitment and grouping of participants into telemedicine and suspended care groups based on study criteria

Overall, 76 patients who resumed face-to-face medical care after the suspension of home visits were included in this study (Fig. 1). Participant characteristics are presented in Table 2. Of the 76 participants, 35 (46.1%) were male, and 41 (53.9%) were female, with a mean age of 74.2 ± 17.4 years. Of these, 20 (26.3%) participants received telemedicine, while 56 (73.7%) did not and had their medical care suspended. None of the telemedicine sessions categorized as “Dentist to Patient” involved the patient alone; a support person such as a spouse, child, caregiver, dietitian, visiting nurse, or speech therapist was always present. Among the 76 participants, fewer patients in the TG lived alone or with only a spouse than those in the SG, with two of the 20 (10%) patients in the TG versus 21 of the 56 (37.5%) in the SG. Of the 23 patients living alone or with a spouse, only 2 requested telemedicine. The frequency of telemedicine use was 1, 2, and 3 sessions for 13, 6, and 1 individuals, respectively. 13 participants were at home during the telemedicine visit, while 7 were in a nursing home for older adults. The most prevalent underlying causes of dysphagia were cerebrovascular diseases (35 cases, 46.0%), followed by neurodegenerative diseases, including dementia and Parkinson’s disease (25 cases, 32.9%). 5 (6.6%) cases were secondary to head injuries, and 11 (14.5%) were classified as being due to other causes. CCI scores were significantly higher in the TG than in the SG. For most other variables, there were no significant differences in participant characteristics between the TG and SG (Table 2). Although not shown in Table 2, one patient in the SG was diagnosed with depression.

**Table 2:** Comparison of participant characteristics between the two groups (n = 76)

Characteristics	Telemedicine group (n = 20)	Suspended group (n = 56)	p-value
Age, median (IQR)	83.0 (66.5–89.5)	76.0 (62.3–86.0)	0.376^a^
Sex, N (%)			
Male	8 (40.0)	27 (48.2)	0.607^b^
Female	12 (60.0)	29 (51.8)	
BMI, mean ± SD	20.2 ± 3.6	18.8 ± 2.8	0.221^c^
BI, median (IQR)	15.0 (0.0–50.0)	0.0 (0.0–40.0)	0.200^a^
Primary disease, N (%)			
Cerebrovascular disease	7 (35.0)	27 (48.2)	0.433^b^
Neurodegenerative disease	7 (35.0)	19 (33.9)	1.000^b^
Head injury	3 (25.0)	2 (3.6)	0.111^b^
Other	3 (25.0)	8 (14.3)	1.000^b^
History of aspiration pneumonia, N (presence) (%)	11 (55.0)	28 (50.0)	0.797^b^
CCI, median (IQR)	2 (2–2)	0 (0–2)	0.002*^,a^
FOIS, median (IQR)	4.00 (2.00–5.00)	3.00 (2.00–4.75)	0.221^a^
DSS, median (IQR)	4.00 (2.25–5.00)	2.00 (3.00–5.00)	0.598^a^
Number of days of interrupted home visits median (IQR)	183.0 (94.5–364.0)	185.5 (140.0–238.0)	0.955^a^
Primary caregiver supporter N (presence) (%)			
None	0 (0)	3 (5.4)	0.562^b^
Spouse	2 (10.0)	18 (32.1)	0.076^b^
Child/Parent/Sibling	11 (55.0)	30 (53.6)	1.000^b^
Home care worker	7 (35.0)	5 (8.9)	0.011*^,b^
Availability of medical professional support, N (presence) (%)	3 (15.0)	12 (21.4)	0.746^b^

SD, standard deviation; IQR, interquartile range; BMI, body mass index; BI, Barthel Index; FOIS, Functional Oral Intake Scale; CCI, Charlson Comorbidity Index; DSS, Dysphagia Severity Scale; Number of days of interrupted home visits: The duration between the last face-to-face medical care before the suspension of home visits and the resumption of face-to-face medical care

*p < 0.05

^a^Mann–Whitney U test

^b^Fisher’s exact test

^c^t test

### Comparison of Adverse Events

The incidence of whole body-related events was 0 (0%, 95% confidence interval: 0.0–16.8) in the TG and 7 (12.5%, 1.2–31.7) in the SG, with no significant difference between groups. However, for dysphagia-related adverse events, 2 (10.0%, 1.2–31.7) cases were observed in the TG, compared to 19 (33.9%, 21.8–47.8) in the SG, indicating significantly fewer cases in the TG than in the SG (p = 0.046). No cases of death, hospitalization, aspiration pneumonia, or choking were observed in the TG. However, two individuals experienced a decline in swallowing function. In contrast, in the SG, 3 individuals died, 4 were hospitalized, 4 had aspiration pneumonia, 3 experienced choking, and 16 experienced a decline in swallowing function. No significant differences were observed for individual outcomes (Table 3).

**Table 3:** Comparison of adverse events between the two groups (n = 76)

Adverse event, N (%) [95% CI]	Telemedicine group (n = 20)	Suspended group (n = 56)	p-value
Whole-body related event	0 (0) [0, 16.8]	7 (12.5) [5.2, 24.1]	0.179
Death	0 (0) [0, 16.8]	3 (5.3) [1.1, 14.9]	0.562
Hospitalization	0 (0) [0, 16.8]	4 (7.1) [1.1, 14.9]	0.568
Dysphagia-related event	2 (10.0) [1.2, 31.7]	19 (33.9) [21.8, 47.8]	0.046*
Aspiration pneumonia	0 (0) [0, 16.8]	4 (7.1) [2.0, 17.3]	0.568
Choking	0 (0) [0, 16.8]	3 (5.3) [1.1, 14.9]	0.562
Decline in swallowing function	2 (10.0) [1.2, 31.7]	16 (28.6) [17.3, 42.2]	0.129

Fisher’s exact test

95% CI, 95% confidence interval

*p < 0.05

### Factors Associated with Dysphagia-Related Adverse Events

No significant differences in baseline characteristics were observed between participants with and without dysphagia-related adverse events (Table 4). Univariate analysis revealed no statistically significant associations between dysphagia-related adverse events and any survey items (Table 5). Binomial logistic regression analysis, adjusting for age, CCI, and DSS as confounders, showed that telemedicine implementation (odds ratio: 0.117, 95% confidence interval: 0.034–0.921, p = 0.040) was significantly associated with fewer dysphagia-related adverse events (Table 6).

**Table 4:** Comparison of patients with and without dysphagia-related events (n = 76)

Characteristics	With dysphagia-related events (n = 21)	Without dysphagia-related events (n = 55)	p-value
Telemedicine implementation			
TG	2 (9.5)	18 (67.3)	0.046*^,b^
SG	19 (90.5)	37 (32.7)	
Age, median (IQR)	82.0 (71.0–86.0)	77.0 (60.0–88.0)	0.393^a^
Sex, N (%)			
Male	8 (38.1)	27 (49.1)	0.448^b^
Female	13 (61.9)	28 (50.9)	
BMI, mean ± SD	19.8 ± 2.9	19.8 ± 2.9	0.392^c^
BI, median (IQR)	5.0 (0.0–45.0)	5.0 (0.0–45.0)	0.682^a^
Primary disease, N (%)			
Cerebrovascular disease	9 (42.9)	25 (45.5)	1.000^b^
Neurodegenerative disease	9 (42.9)	17 (30.9)	0.419^b^
Head injury	1 (4.8)	4 (7.3)	1.000^b^
Other	2 (9.5)	9 (16.4)	0.717^b^
History of aspiration pneumonia, N (presence) (%)	11 (52.4)	28 (50.9)	1.000^b^
CCI, median (IQR)	2 (2–2)	2 (0–2)	0.519^a^
FOIS, median (IQR)	3 (2–5)	3 (2–5)	0.592^a^
DSS, median (IQR)	4 (3–5)	3 (3–5)	0.919^a^
Number of days of interrupted home visits median (IQR)	187.5.0 (155.8–234.5)	183.0 (113.8–264.8)	0.646^a^
Primary caregiver supporter N (presence) (%)			
None	1 (4.8)	2 (3.6)	1.000^b^
Spouse	5 (23.8)	15 (27.3)	1.000^b^
Child/Parent/Sibling	11 (52.4)	30 (54.5)	1.000^b^
Home care worker	4 (19.0)	8 (14.5)	0.727^b^
Availability of medical professional support, N (presence) (%)	4 (15.0)	11 (21.4)	1.000^b^

SD, standard deviation; IQR, interquartile range; TG, Telemedicine group; SG, Suspended group; BMI, body mass index; BI, Barthel Index; FOIS, Functional Oral Intake Scale; CCI, Charlson Comorbidity Index; DSS, Dysphagia Severity Scale; Number of days of interrupted home visits: The duration between the last face-to-face medical care before the suspension of home visits and the resumption of face-to-face medical care

*p < 0.05

^a^Mann–Whitney U test

^b^Fisher’s exact test

^c^t test

**Table 5:** Results of simple regression analysis of dysphagia-related events (n = 76)

Characteristics	p-value	Odds Ratio (95% CI)
Sex	0.391	1.567 (0.561, 4.377)
Primary caregiver: Home care worker	0.631	1.382 (0.368, 5.186)
Primary caregiver supporter: None	0.822	1.325 (0.114, 15.431)
Primary disease: Neurodegenerative disease	0.716	1.250 (0.376, 4.159)
Primary disease: Cerebrovascular disease	0.866	1.091 (0.398, 2.988)
History of aspiration pneumonia	0.909	1.061 (0.388, 2.902)
BMI	0.387	1.084 (0.903, 1.300)
Age	0.205	1.021 (0.989, 1.055)
Number of days of interrupted home visits	0.850	1.000 (0.996, 1.005)
BI	0.882	0.999 (0.981, 1.017)
Availability of medical professional support	0.926	0.941 (0.263, 3.365)
DSS	0.710	0.930 (0.634, 1.364)
Primary caregiver: Child/Parent/Sibling	0.866	0.917 (0.335, 2.511)
CCI	0.697	0.914 (0.581, 1.437)
FOIS	0.465	0.889 (0.648, 1.219)
Primary caregiver: Spouse	0.759	0.833 (0.260, 2.675)
Primary disease: Head injury	0.695	0.638 (0.067, 6.057)
Other (Primary disease)	0.454	0.538 (0.106, 2.726)
Presence of telemedicine implementation	0.055	0.216 (0.045, 1.032)

95% CI, 95% confidence interval; BMI, body mass index; Number of days of interrupted home visits: The duration between the last face-to-face medical care before the suspension of home visits and the resumption of face-to-face medical care; BI, Barthel Index; DSS, Dysphagia Severity Scale; CCI, Charlson Comorbidity Index; FOIS, Functional Oral Intake Scale

*p < 0.05

**Table 6:** Results of binomial logistic regression analysis of dysphagia-related events (n = 76)

	Odds ratio	95% CI	p-value
Presence of telemedicine implementation			
Suspended group	1.00		0.040*
Telemedicine group	0.177	(0.034, 0.921)	
Age	1.024	(0.989, 1.060)	0.179
CCI	1.044	(0.646, 1.687)	0.861
DSS			
Scores 1–4	1.00		0.516
Scores 5–7	1.483	(0.452, 4.865)	

95% CI, 95% confidence interval; CCI, Charlson Comorbidity Index; DSS, Dysphagia Severity Scale; DSS is a seven-point scale: scores 1–4 indicate aspiration present, and 5–7 indicate aspiration absent

*p < 0.05

## Discussion

### Access to and Support for Telemedicine

Even if HMC for patients with dysphagia is suspended, fewer dysphagia-related adverse events were observed among those who chose to continue care via telemedicine than among those who chose to suspend care. This finding suggests that telemedicine can serve as an effective alternative for providing dysphagia care and is preferable to discontinuing care entirely.

In this study, among patients with dysphagia who temporarily suspended medical care, 7.1% were hospitalized, and another 7.1% developed aspiration pneumonia. While there are no prior studies directly reporting hospitalization or aspiration pneumonia rates in patients with dysphagia under home care during suspended care periods, one study reported that 48.3% of 178 older adults receiving home care experienced unplanned hospitalizations over a 4-year follow-up period [22]. Another study involving 689 patients with dysphagia found that 22% developed aspiration pneumonia over a 2-year follow-up period [23]. These findings suggest that prolonged suspension of care could lead to higher rates of adverse events, consistent with trends observed in this study. A significant difference was found in CCI scores between patients who opted for telemedicine and those who suspended care, with the TG having more comorbidities associated with increased mortality. This indicates that patients may have chosen telemedicine owing to a perceived heightened risk of dysphagia-related adverse events during the suspension of face-to-face care. Most participants were older adults aged ≥ 65 years requiring nursing care, while younger participants required support owing to disabilities. Only a few participants were fully independent in their daily lives. In this study, patients who received telemedicine were supported by medical professionals, family members, or home caregivers, and there were no cases where patients were alone during sessions. In this study, healthcare professionals were present during telemedicine sessions in only 3 of the 20 cases. This limited involvement was due to restrictions imposed by the COVID-19 pandemic, which prevented other healthcare professionals from having direct contact with patients. Patients often declined online medical care if a family member, such as a child or caregiver, or a medical professional was not present during home visits. Barriers to implementing telemedicine included a lack of ICT equipment, limited understanding of ICT, and insufficient support for telemedicine use. To facilitate the broader adoption of telemedicine, improving ICT equipment and establishing accessible, user-friendly communication environments, such as through smartphones, is essential. Lending ICT devices from medical institutions and providing guidance on their operation or incorporating ICT education for older adults and caregiving families as part of preventive care initiatives could significantly promote telemedicine adoption.

Telemedicine also offers the potential for improving interprofessional collaboration by enabling the sharing of information among healthcare providers who may face challenges meeting in person owing to time or distance constraints. Once ICT equipment is optimized, telemedicine could reduce the frequency of home visits and the overall time required for medical treatment, increasing convenience for both patients and clinicians [24]. In the future, developing a comprehensive system for home healthcare that integrates telemedicine should be prioritized to enhance access and efficiency.

### Clinical Utility of Telemedicine

Telemedicine enables external assessments of a patient’s facial expressions, posture, and daily eating environment, as well as communication through conversation. It is also useful for evaluating a patient’s oral condition and function [25]. Another advantage is that it prevents patients from concentrating on eating because they are distracted by visitors.

Additionally, allowing patients to undergo rehabilitation in their daily lives can increase adherence to and motivation for rehabilitation [26, 27]. However, telemedicine has limitations, as it cannot assess swallowing function using tools such as swallowing endoscopes. Nevertheless, it enables healthcare professionals to verify whether patients and caregivers are practicing techniques taught during in-person visits, such as adjusting dietary forms and implementing compensatory strategies. In this study, we believe that continued intervention without suspension of dysphagia rehabilitation may have contributed to maintaining patient and caregiver adherence, which may have reduced the number of dysphagia-related adverse events in the TG.

Dysphagia rehabilitation involves not only examination and training but also ongoing follow-up to prevent complications such as aspiration pneumonia, malnutrition, and choking. For patients with a history of silent aspiration or choking, those requiring advanced diagnostic examinations such as swallowing endoscopy or videofluorography, or those practicing oral intake while at high risk of aspiration, telemedicine assessments may be insufficient. Therefore, the effectiveness of telemedicine depends on the severity of dysphagia, and clinicians providing telemedicine services should be adequately trained and prepared [28]. Clinicians should possess sufficient knowledge of dysphagia rehabilitation, including the ability to assess risks, determine the need for face-to-face care, and provide appropriate instructions in emergency situations. In the future, it will be important to standardize the qualifications of clinicians, such as requiring certification in dysphagia rehabilitation approved by professional societies. In this study, dentists providing telemedicine had substantial clinical experience, having delivered face-to-face care before the suspension of home visits and possessing detailed knowledge of each patient’s condition. All patients were reexamined and underwent at least one thorough assessment via a swallowing endoscope. Consequently, telemedicine consultations were tailored to the patient’s prior condition and characteristics. This enabled appropriate risk assessment and prevention of adverse events. Telemedicine consultations were conducted similarly to face-to-face consultations, with instructions provided to the patient or telemedicine assistant. No issues were reported in any of the cases. Telemedicine served as a complementary measure when face-to-face care was not feasible. While face-to-face care, including assessments such as swallowing endoscopy or videofluorography, remains invaluable, telemedicine provides an effective alternative when direct medical care is inaccessible.

### Challenges of Telemedicine in Dysphagia Rehabilitation

Face-to-face medical care is not always necessary for dietary observation and swallowing training instructions in dysphagia rehabilitation, which is considered highly compatible with telemedicine. The utility of telemedicine has been demonstrated in assessing dysphagia in hospitalized patients with acute cerebrovascular disease [4], monitoring nutritional disorders and dysphagia after head and neck cancer surgery [29–31], and observing the dietary habits of pediatric patients with cerebral palsy receiving home care [32]. Telemedicine can serve as an alternative to face-to-face medical care, particularly in situations where access to dysphagia rehabilitation is challenging. The findings of this study suggest that telemedicine is a valuable tool for supporting patients with dysphagia receiving medical care at home or in nursing homes. Telemedicine is also advantageous in emergencies that impede in-person visits or in remote areas with limited access to specialized medical care.

However, telemedicine faces several challenges, including issues related to medical billing and insurance systems, insufficient telehealth infrastructure, and inadequate clinician training, which contribute to its perception as a secondary service compared to face-to-face care [8, 33]. Collecting medical information via a screen can also be challenging, particularly for physical findings that require in-person assessment in a home setting. The presence of family doctors, dentists, or other healthcare professionals on the patient’s side during telemedicine sessions could facilitate information sharing and enable telemedicine to complement initial medical examinations. Additionally, their involvement improves safety by addressing unexpected situations or emergencies that may arise during telemedicine consultations. Telemedicine is a valuable complement to face-to-face care and has the potential to enhance community healthcare. Increased collaboration among healthcare professionals will broaden the indications for telemedicine and improve its effectiveness in dysphagia rehabilitation.

### Limitations

The relationship between telemedicine use and the incidence of adverse events was clarified in this study. However, as a retrospective study, the causal relationship between specific aspects of telemedicine, such as frequency, duration, and the nature of patient instructions, and the incidence of adverse events, remains unclear. Most patients with dysphagia in this study required a caregiver, and the implementation of telemedicine care may have been influenced not only by the patient’s self-selection but also by the presence of a supportive environment enabling telemedicine use. Therefore, the caregiver support system may have introduced bias into whether patients participated in telemedicine. Additionally, the SG included 12 medical professional supporters; however, the impact of co-interventions performed by these professionals was not evaluated. The small sample size in this study limited the statistical evaluation of individual adverse events. For all adverse events, the TG demonstrated a lower incidence rate than the SG. Among dysphagia-related adverse events, there were no cases of severe events such as aspiration or choking; only two relatively mild events, specifically a decline in swallowing function, were observed in the TG. While differences were observed when adverse events were analyzed collectively, the small sample size limits the ability to draw definitive conclusions regarding global effects. Increasing the sample size is essential for further validation. Moreover, the limited number of patients in the TG and the lack of adequate data on disease stage and duration for systemic diseases and dysphagia hindered the adjustment for confounding factors. The lack of difference in the incidence of systemic-related events between groups may be owing to the shorter duration of the interruption of home visits. To improve the quality and effectiveness of telemedicine, future studies should prospectively investigate telemedicine interventions in greater detail, particularly focusing on their extent and impact.

## Conclusion

During the COVID-19 pandemic, the incidence of dysphagia-related adverse events was significantly reduced by continuing dysphagia rehabilitation interventions via telemedicine, even amidst the suspension of home visits and medical care for patients with dysphagia. Telemedicine proved to be a valuable alternative when face-to-face care was unavoidably interrupted.

## Data Availability

The datasets generated during and analyzed during the current study are available from the corresponding author upon reasonable request.
